# Physical attractiveness and women’s intra-household bargaining power

**DOI:** 10.3389/fpsyg.2022.853083

**Published:** 2022-11-14

**Authors:** Zhongwu Li

**Affiliations:** School of Economics, Zhejiang University of Technology, Hangzhou, China

**Keywords:** physical attractiveness, intra-household bargaining power, self-esteem, generalized structural equation model, CFPS

## Abstract

This paper explores the role of physical attractiveness in affecting women’s intra-household bargaining power. The empirical analysis based on the China Family Panel Studies finds that physical attractiveness significantly increases women’s intra-household bargaining power. To solve the endogenous problem of physical attractiveness, we employ an instrument-variable-based regression to corroborate the conclusion. Using generalized structural equation model, we show that income, self-esteem, and interpersonal relationship are three possible transmission channels (or mediators) between physical attractiveness and women’s intra-household bargaining power.

## Introduction

Physical attractiveness or beauty is the degree to which a person’s physical features are considered esthetically pleasing or beautiful (Dion et al., 1972). It is mainly determined by both constant and changing components. The constant component has relatively stable physical characteristics, such as facial features and body shape (Sainani, 2015). The changing component always changes with the specific situation, that is, changes in grooming, dress, makeup, body posture, and facial expression (Wong and Penner, 2016). Although the constant component of physical appearance is largely determined by parents’ genes, the maintenance and modification of the changing component can greatly improve a person’s physical attractiveness. The love for beauty is an eternal topic among women in China (Hua, 2009, 2013). According to a research report,^1^ female consumers in China occupy more than half of the online shopping market in 2018, among which beauty and skin care consumption has the leading position. Many women even spend large amounts of money trying plastic surgery to make their appearance more in line with the public’s esthetic standards in China (Waiyee, 2021).

Due to the stereotype of “to be beautiful is to be good (halo effect),” physical appearance often influences individual’s social evaluations on other people. For example, in terms of health evaluation, Tsiga et al. (2016) indicated that doctors have more positive evaluations on patients who are physically attractive. In the marriage market, good-looking people are favored on TV dating shows, and are more likely to get a better-looking partner (Lee et al., 2008). In the labor market, attractive job seekers are more likely to be interviewed and hired by a company (Deng et al., 2020). Further, they are more likely to be recognized by their colleagues when they give their opinions, which in turn leads to more income (Hosoda et al., 2003). Previous studies have found that this “to be beautiful is to be good” bias is largely caused by the halo effect, i.e., one-sided attributions of good personality qualities to physically attractive people (Lai et al., 2013). Beautiful individuals are subconsciously believed to have some valuable personality traits, such as trustworthiness, friendliness, helpfulness, and intelligence, and this stereotype is self-fulfilling (Langlois et al., 2000). In addition, evolutionary biologists have proposed the “good genes” theory, emphasizing that the genes that make a person beautiful also make a person intelligent and sociable, namely the phenomenon of “good genes clustering” (Brand et al., 2012). As the research of Kanazawa (2011) showed that, people who are physically attractive do tend to be more intelligent in life and work, as well as more creative.

Considering that physically attractive individuals enjoy advantages in terms of intelligence, confidence, and popularity (Grammer et al., 2003; Lai et al., 2013), many researchers claim that there is a beauty premium, namely, physical attractiveness pays off in economic and political affairs (Mobius and Rosenblat, 2006; Jin et al., 2017; Póvoa et al., 2020). Attractive people achieve better outcomes than their less attractive counterparts, whether in economic cooperation or political elections (Andreoni and Petrie, 2008; Peng et al., 2020). There have been a series of studies on the “beauty premium,” such as beauty and employment (Deng et al., 2020), beauty and entrepreneurship (Baron, 2000), and beauty and happiness (Datta Gupta et al., 2016). Contrastly, few studies have examined the relationship between beauty and women’s empowerment in the family, furthermore, related studies are almost exclusively in Western developed countries (e.g., Dilmaghani, 2021 in Canada; Esping-Andersen and Schmitt, 2019 in German; Oreffice and Quintana-Domeque, 2012 in United States). However, the existing literature reveals that both standards of beauty and intra-household bargaining process are heavily influenced by culturally shaped norms, customs, and expectations (Agarwal, 1997; Madan et al., 2018). So, it is needed to study the beauty effect in women’s empowerment within family in some non-Western developing countries. The contemporary China is a good case for the study given the background of serious gender imbalance, i.e., 30 million more men than women in China. So, beautiful women can stand out in the marriage and love market through their good looks, and become the favorite marriage partners of many men with favorable family backgrounds. In marriage, many men will feel honored by the good looks of their wives, thereby possibly ceding part of intra-household bargaining power to their wives, and even heeding to wives’ wishes and preferences (Esping-Andersen and Schmitt, 2019). Additionally, physical attractiveness might increase wives’ threat point in the intra-household bargaining game because the attractive wives can re-enter the marriage market easily in case of divorce. However, these are some untested observations from our daily life, but there have been no relevant empirical studies on this found. Specifically, we have no idea of whether beauty can improve women’s intra-household bargaining power in a national representative sample.

### The present study

In view of this, under the mainstream resource-agency-achievement empowerment framework (see Kabeer, 1999 for details), this paper uses the CFPS dataset to study the impact of women’s beauty on their intra-household bargaining power in China. Among them, the initial investment of resources is the beauty of women, which is regarded as one of the most important assets of women. The main agency, which is the ability of individuals to use resources to achieve expected achievements. The achievement of empowerment is women’s intra-household bargaining power. Additionally, we propose some new mediating channels (e.g., self-esteem) for future researchers to study how a factor influences women’s intra-household bargaining power. Further, this paper quantifies the direct, indirect, and mediating effects of three mediating variables by using the generalized structural equation model. And thus, more targeted, and accurate policy recommendations can be put forward.

## Conceptual framework and hypothesis

Based on the existing literature (Major et al., 1984; Haas and Gregory, 2005; Scholz and Sicinski, 2015), we propose that physical attractiveness affects women’s intra-household bargaining power mainly through the following three transmission channels (or mediators): income, self-esteem, and interpersonal relationship.

### Conceptual framework

#### Physical attractiveness–income–women’s intra-household bargaining power

On the one hand, existing studies have shown that physical attractiveness is an important factor in promoting individual income (Scholz and Sicinski, 2015; Anýžová and Matějů, 2018). In their groundbreaking study, Hamermesh and Biddle (1994) found that beauty premium in the US labor market is as high as 10–15 percent, comparable to the wage gap caused by race and gender.^2^ Since then, there have been a series of studies on the premium of beauty in the labor market (Judge et al., 2009; Lynn, 2009). For example, Wong and Penner (2016) used the US data to study the relationship between physical attractiveness and income, and found that the income gap between attractive individuals and average ones is even as high as 20%. Meanwhile, many Chinese scholars have studied the impact of beauty on personal income, and found that there are also obvious phenomena of beauty premium and ugliness penalty in China’s labor market (Gu and Ji, 2019; Peng et al., 2020). For example, Peng et al. (2020) showed that good-looking individuals earn roughly 5.4% more than the rest, and bad-looking individuals earn roughly 3.3% less than the rest. On the other hand, research shows that the quantity of resources such as income and assets will greatly influence individual’s bargaining power in the family decision-making process (Mabsout and van Staveren, 2010; Martínez, 2013). More income means a stronger threat point in the bargaining game in case of marriage failures, so, it is positively associated with one’s intra-household bargaining power (Wang et al., 2020). Therefore, physical attractiveness might increase women’s income, which subsequently could improve women’s intra-household bargaining power.

#### Physical attractiveness–self-esteem–women’s intra-household bargaining power

Studies have shown that physical attractiveness enhances individual self-esteem (Major et al., 1984; Tran et al., 2020). Individuals who are physically unattractive may be viewed negatively by others, even experience invisible psychological discrimination, which can lead to feelings of depression, stress, and low self-esteem (Frieze et al., 1990). In sharp contrast, physically attractive individuals receive more attention and positive evaluations from others, which might enhance a person’s sense of self-acceptance and self-esteem (Thornton and Ryckman, 1991; Patzer, 2006). For example, Ivtzan and Moon (2008) revealed that participants in the high attractiveness group score significantly higher on seven of the 12 self-actualization scales compared to the participants in the low attractiveness group. Meanwhile, Bénabou and Tirole (2002) pointed out that those high in self-esteem can present themselves positively to others easily, and are more motivated to persevere in pursuing their goals in face of setbacks and temptations. Due to the benefits of positive self-esteem, it has been found that self-esteem has a significantly positive effect on individual career status and salary, marriage, academic performance, and even happiness (Diener and Diener, 2009; Orth and Robins, 2014). Furthermore, a study revealed that self-esteem can improve women’s intra-household bargaining power at the psychological level (Li and Wang, 2020). Therefore, physical attractiveness might enhance women’s self-esteem, which subsequently could improve women’s intra-household bargaining power.

#### Physical attractiveness–interpersonal relationship–women’s intra-household bargaining power

Previous research has shown that beauty will broaden individual network of relationships (Gordon et al., 2013; O’Connor and Gladstone, 2018). In interpersonal communication, the first features to be recognized are a person’s appearance and body shape. There is often a stereotype that the talents and interpersonal skills of the unattractive are inferior to those of the attractive (Jackson et al., 1995; Kanazawa, 2011). As a result, people react positively to attractive people and negatively to unattractive people (Tartaglia and Rollero, 2015). This allows attractive people to gain more communication opportunities and confidence, which will further improve their interpersonal, communication, and negotiation skills (Mobius and Rosenblat, 2006). This could largely increase women’s networks of relationships in their communities and workplaces. In the person-to-person trust game, attractive individuals are more likely to be trusted by others (Eckel and Wilson, 2004), which increases the likelihood that others will cooperate with those more attractive individuals. Meanwhile, social capital based on trust and cooperation could significantly improve women’s relative fallback position in intra-household bargaining process (Mayoux, 2001; Eklund et al., 2007). Therefore, physical attractiveness might increase women’s interpersonal relationship, which subsequently could enhance women’s intra-household bargaining power.

**Figure 1:**
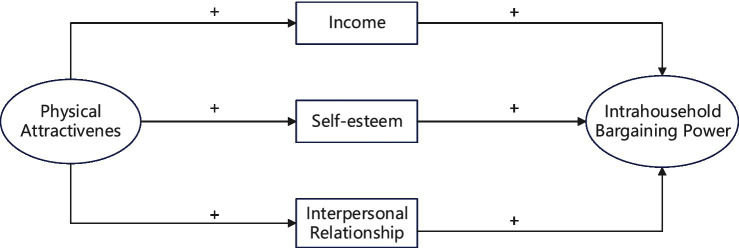
The proposed conceptual framework for physical attractiveness to influence women’s intra-household bargaining power.

As shown in Figure 1, physical attractiveness might improve women’s income, self-esteem, and interpersonal relationship, which, in turn, increase their intra-household bargaining power. It is worth noting that although the paper proposes that income, self-esteem, and interpersonal relationship may be the mediating mechanisms through which physical attractiveness affects women’s intra-household bargaining power, it does not mean that these three possible influencing mechanisms are sufficient to explain the impact of physical attractiveness on women’s intra-household bargaining power. In addition to these three variables, there may be other mechanisms at play at the same time, and this paper is still an exploratory study.

#### Hypothesis

From the conceptual framework, we propose that physical attractiveness might have some impacts on women’s intra-household bargaining power, due to its strong role in raising women’s income level, and enhancing self-esteem and interpersonal relationship. Based on this, this paper proposes the following research hypotheses:

*H1*: Physical attractiveness can improve women’s intra-household bargaining power.

*H2*: Income, self-esteem, and interpersonal relationship are three important transmission channels for physical attractiveness to improve women’s intra-household bargaining power.

## Materials and methods

### Data

We conduct the empirical analysis using a nationally representative dataset from the China Family Panel Studies (CFPS, it is publicly accessible through https://www.isss.pku.edu.cn/cfps/en/index.htm). The CFPS is a biennial national survey conducted by the Institute of Social Sciences at Peking University in collaboration with the University of Michigan’s Survey Research Center. The CFPS seeks to provide the most comprehensive microdata for academic research and public policy analysis by tracking indicators at the individual, family, and community levels. While starting from 2010, several rounds of surveys have been conducted to track social, economic, demographic, educational, and health changes in contemporary China (Xie and Hu, 2014; Xie and Lu, 2015). So far, the latest CFPS survey has been updated to 2018, however, only the 2012 CFPS survey contains key variables needed in the paper, including respondent’s intra-household bargaining power, self-esteem, and other socioeconomic characteristics. Even though the 2012 CFPS survey has no advantages over later rounds of surveys in timeliness, our findings could lay the foundations for future search in related fields.

### Sample

To investigate the determinants of women’s intra-household bargaining power, so, we constrain the survey sample to women in marriage. There are totally 9,939 observations available in the 2012 CFPS adult, family economy, and family relationship datasets. Meanwhile, to detect a causal effect of women’s physical attractiveness on their bargaining power, the paper tries to find an appropriate instrumental variable in the 2010 CFPS adult dataset. So, we have to merge the 2010 CFPS adult dataset with the 2012 CFPS adult, family economy, and family relationship datasets by using the personal identification code (PID).^3^ Finally, there remain 7,220 observations in the survey sample. After deleting the variables with missing values and non-response items, we get 6,728 observations in analytical sample for regression analysis. According to our balance test, the variables used in the study have similar means and SDs between the analytical sample and the survey sample. So, the missing values and influential points would not introduce a significant bias.

### Measures

#### Dependent variable

The dependent variable, Bargain_power, is the intra-household bargaining power in major family affair. It is assessed by the question of “Who is the decision maker of major family affairs including household expenditure allocation, household investment and savings, house purchase and construction, high-priced consumer goods?” If the respondent answers “husband,” it is assigned with 1, “negotiation together” 2, and “wife” 3. There will be disagreements between the couple in answering the above questions, that is, their respective answers contradict the actual situation in the family. For example, the husband (/wife) is the decision-maker of major family affairs, but they answer that the wife (/husband) is the decision-maker of family affairs. This makes it difficult for empirical estimates, so, seven of these contradictory observations are deleted.

To measure women’s intra-household bargaining power objectively in multiple dimensions, this paper also uses the household financially responsible person (Finance_power) as the alternative indicator in robustness check. In the CFPS 2012, it is assessed by the question of “Who is in charge of household finance?” Like the variable of Bargain_power, if the respondent answers *husband*, it is 1, *negotiation together* 2, and *wife* 3.

#### Key independent variable

The key independent variable, women’s physical attractiveness (Appearance), is obtained by interviewers to evaluate the respondent’s appearance at the end of the interview. The interviewers are asked to rate the respondent’s physical appearance on a scale of 1–7, with 1 being *very unattractive* and 7 being *very attractive*. So, a higher rating indicates the respondent is more attractive. In a series of studies on physical appearance effect, scholars have often used this indicator to measure physical attractiveness (e.g., Peng et al., 2020). The main reason is that, in a specific region and time point, groups with similar cultural backgrounds and preferences have very similar criteria for judging beauty (Hatfield and Sprecher, 1986). This standard of beauty also changes slowly over time. Therefore, the homogeneity and stability of esthetic characteristics provide a theoretical basis for us to measure and compare the appearance of respondents.

#### Mediating variables

In the paper, we have three mediating variables, including annual income (Income, unit: 10,000 RMB), self-esteem (Self_esteem), and interpersonal relationship (Relation). Among them, interpersonal relationship (Relation) is assessed by the question of how is the interpersonal relationship between you and others. The respondent has five options corresponding with 1–5, and a larger value indicates better interpersonal relationship. Self_esteem is constructed by the Rosenberg Self-esteem Scale Survey (in short, RSES). As the most common and reliable measure of self-esteem, the RSES is widely used in psychology and economics (Bowles et al., 2001; Heckman et al., 2006). Considering data availability in the CFPS, we draw on the practice of Orth et al. (2008) to measure women’s self-esteem by using a simplified version of the RSES. This is not unusual. In an important review, Gray-Little et al. (1997) pointed out that the 10-item RSES “could be shortened without comprising the measurement of global self-esteem.” In psychology literature, Tafarodi and Swann (1995) used six items in the RSES, and only two items between their study and the RSES overlapped. We construct a score which is like the RSES score by four statements of self-approval and disapproval:(A) I feel as good as others, (B) I feel I am a failure, (C) I feel I cannot go on with my life, and (D) I am hopeful about the future. The respondent is given four options for each of these statements: *strongly agree*, *agree*, *disagree*, and *strongly disagree*. We give positive ratings to statements (A) and (D) that tend to be self-approval, and negative ratings to statements (B) and (C) that tend to be self-disapproval. For positive ratings, 1 is assigned to *strongly disagree*, 2 to *disagree*, 3 to *agree*, and 4 to *strongly agree*. For negative ratings, it is scored in a reverse manner.

Then, the scores obtained from the four statements are added together to construct an ordinal self-esteem variable. The higher the score is, the stronger sense of self-esteem is. Cronbach’s alpha in this study is 0.79.

#### Instrument variable^4^

To detect any causal relationship between physical attractiveness and women’s intra-household bargaining power, we employ an instrument-based approach to cut off any possible confounding paths. Finding a valid instrument for physical attractiveness is difficult in related studies (Hamermesh and Abrevaya, 2013). We follow Hamermesh and Abrevaya (2013) to use the lagged physical attractiveness measurement (2 years ago) as the instrument for physical attractiveness in the year explored in the paper. That is, we select physical attractiveness in 2010 (Appearance_2010) as the instrument variable of physical attractiveness in 2012. It is measured in the same way as that of Appearance. The motivation for using physical attractiveness in 2010 as the instrument variable is as follows. On the one hand, the time span of 2 years is relatively short, so, the appearance of the respondent in these 2 years is highly correlated. It satisfies the condition of correlation of instrument variable with the endogenous variable. On the other hand, the respondent’s physical appearance in 2010 will not directly affect their intra-household bargaining power in 2012, and vice versa, thus satisfying the condition of exclusion restriction of instrument variable.

#### Covariates

Based on literature on intra-household bargaining power (Agarwal, 1997; Wang, 2014; Baland and Ziparo, 2018), this paper controls for some important bargaining power-influencing factors in the multivariate analysis. Among them, variables at the individual level include household registration (Urban), years of education (Education), age (Age), annual income (Income, unit: 10,000 RMB), interpersonal relationship (Relation), years of marriage (Marriage_year), and intimate relationship with your spouse (Intimacy). Among them, Intimacy is assessed by the question of how important is it for you to have an intimate relationship with your spouse. The respondent has five options (1–5), and a larger value indicates it is more important to have an intimate relationship with spouse. Characteristic variables within the family include spouse’s physical attractiveness (S_Appearance), years of education (S_Education), age (S_Age), annual income (S_Income, unit: 10,000 RMB), and number of children (Nchild). Mabsout and van Staveren (2010) showed that community environment in which an individual lives, such as norms, beliefs, and traditions, will affect women’s intra-household bargaining power. For this reason, we use attitudes toward traditional family ethics of ‘people should carry on the family line’ in community as an environmental variable. The detailed definitions of variables are listed in Table 1.

**Table 1:** Definitions of main variables.

Variables	Definitions
*Bargain_power*	Who is the decision-maker of major family affairs? (1 = husband, 2 = negotiation together, 3 = wife)
*Finance_power*	Who takes charge of household finance? (1 = husband, 2 = negotiation together, 3 = wife)
*Appearance*	Physical attractiveness rated by the interviewers (1–7, a larger value indicates more physical attractiveness)
*S_Appearance*	Spouse’s physical attractiveness rated by the interviewers (1–7, a larger value indicates more physical attractiveness)
*Urban*	The household registration type (1 = urban household registration, 0 = rural household registration)
*Education*	Years of education (0–22)
*S_Education*	Years of spouse’s education (0–22)
*Age*	Age (18–87)
*S_Age*	Spouse’s age (19–90)
*Income*	Annual income (unit: 10,000 RMB)
*S_Income*	Annual income of spouse (unit: 10,000 RMB)
*Relation*	How is the interpersonal relationship between you and others, self-reported one (1–5, a larger value indicates better interpersonal relationship)
*S_Relation*	How is the interpersonal relationship between your spouse and others (1–5, a larger value indicates better interpersonal relationship of your spouse)
*Marry_year*	Years of marriage (0–64)
*Nchild*	Number of children (0–9)
*Intimacy*	How important is it for you to have an intimate relationship with spouse, self-reported one (1–5, a larger value indicates more importance)
*Family_linec*	Attitudes toward traditional family ethics of “people should carry on the family line” in community (1–5, larger value indicates more traditional community)
*Self_esteem*	A larger value indicates a stronger sense of self-esteem and self-acceptance (4–16)
*Appearance_2010*	Physical attractiveness rated by the interviewers in 2010 (1–7, a larger value indicates more physical attractiveness)

Additionally, the data in this paper came from a questionnaire survey, and the evaluation of respondent’s physical appearance comes from the interviewers’ observations. Considering that seasonal changes may affect the accuracy of interviewers’ judgments, this paper uses month effect to control for such seasonal heterogeneities. Different provinces in China have different regional cultures, with some places giving direct comments on others’ looks while others are more euphemistic. Additionally, provinces have different levels of economic developments. Regarding this, provincial dummies are added to control for the impact of such regional differences.

### Data analysis strategy

We start the analyses by displaying the descriptive statistics of major variables (Table 2) and cross-tabulation of women’s physical attractiveness and their intra-household bargaining power (Table 3).

**Table 2:** Descriptive statistics of main variables.

	*N*	Mean	*SD*	Min	Median	Max
*Bargain_power*	6,728	1.50	0.75	1	1	3
*Finance_power*	6,728	1.91	0.92	1	2	3
*Appearance*	6,728	5.15	1.21	1	5	7
*S_Appearance*	6,728	5.21 7	1.18	1	5	7
*Urban*	6,728	0.44	0.50	0	0	1
*Education*	6,728	5.81	4.62	0	6	22
*S_Education*	6,728	7.66	4.11	0	9	22
*Age*	6,728	48.51	12.54	18	48	87
*S_Age*	6,728	50.48	12.86	19	49	90
*Income*	6,728	0.59	1.40	0	0.20	80
*S_Income*	6,728	1.30	1.97	0	0.86	80
*Relation*	6,728	4.00	0.84	1	4	5
*S_Relation*	6,728	3.97	0.86	1	4	5
*Marry_year*	6,728	24.01	13.14	0	24	64
*Nchild*	6,728	1.30	0.88	0	1	9
*Intimacy*	6,728	4.34	0.88	1	5	5
*Family_linec*	6,728	4.04	0.46	1	4.10	5
*Self_esteem*	6,728	12.16	2.07	4	12	16
*Appearance_2010*	6,728	4.90	1.21	1	5	7

N, number of observations; Mean, mean value; SD, standard deviation; Min, minimum value; Median, median value; and Max, maximum value.

**Table 3:** Cross-tabulation of physical attractiveness and women’s intra-household bargaining power.

*Appearance*	*Bargaing_power* = 1	Percentage %	*Bargaing_power* = 3	Percentage %
1	14	82.35	0	0.00
2	89	78.07	8	7.02
3	387	74.14	69	13.22
4	949	66.83	221	15.56
5	1,432	67.14	335	15.71
6	1,361	66.68	316	15.48
7	555	58.73	201	21.27
Total	4,787	66.56	1,150	15.99

Bargaing_power = 1, husband is the decision-maker of major family affairs; Bargaing_power = 3, wife is the decision-maker of major family affairs; In order to better compare the difference in the percentage of decisions made by men and women in major family affairs, we do not list ‘negotiation together.’

In the second step, given the ordinal nature of the dependent variable, we employ ordinal probit model to estimate the relationship between physical attractiveness and women’s intra-household bargaining power (Table 4). Meanwhile, the ordinary least squares (OLS) method is employed as the reference regression. Specifically, taking women’s intra-household bargaining power in major family affairs as the dependent variable, women’s physical attractiveness as the independent variable, this paper establishes the following econometric model to test their relationship:


(1) Bargain_power=β∗Appearance+γ∗X+μ

**Table 4:** The impact of physical attractiveness on women’s intra-household bargaining power.

	OLS		Ordinal probit	
	(1)	(2)	(3)	(4)
*Appearance*	0.04^***^	0.04^***^	0.07^***^	0.07^***^
	(0.01)	(0.01)	(0.02)	(0.02)
*S_Appearance*	−0.04^***^	−0.03^***^	−0.07^***^	−0.06^***^
	(0.01)	(0.01)	(0.02)	(0.02)
*Urban*	0.10^***^	0.09^***^	0.17^***^	0.15^***^
	(0.02)	(0.02)	(0.03)	(0.04)
*Education*	0.03^***^	0.03^***^	0.05^***^	0.05^***^
	(0.00)	(0.00)	(0.00)	(0.00)
*S_Education*	−0.02^***^	−0.02^***^	−0.03^***^	−0.03^***^
	(0.00)	(0.00)	(0.00)	(0.00)
*Age*	−0.00	−0.00	−0.00	−0.00
	(0.00)	(0.00)	(0.01)	(0.01)
*S_Age*	0.00	0.01^*^	0.01	0.01
	(0.00)	(0.00)	(0.01)	(0.01)
*Income*	0.02^***^	0.02^***^	0.04^***^	0.03^***^
	(0.01)	(0.00)	(0.01)	(0.01)
*S_Income*	−0.01^**^	−0.01^***^	−0.02^**^	−0.03^***^
	(0.01)	(0.01)	(0.01)	(0.01)
*Relation*	0.03^***^	0.03^***^	0.05^**^	0.05^**^
	(0.01)	(0.01)	(0.02)	(0.02)
*S_Relation*	−0.02^**^	−0.02^**^	−0.04^**^	−0.04^*^
	(0.01)	(0.01)	(0.02)	(0.02)
*Marry_year*	−0.00^*^	−0.00^*^	−0.01	−0.01
	(0.00)	(0.00)	(0.00)	(0.00)
*Nchild*	−0.04^***^	−0.04^***^	−0.09^***^	−0.08^***^
	(0.01)	(0.01)	(0.02)	(0.02)
*Intimacy*	−0.03^***^	−0.03^***^	−0.05^**^	−0.05^***^
	(0.01)	(0.01)	(0.02)	(0.02)
*Family_linec*	−0.07^***^	−0.05^**^	−0.11^***^	−0.07^*^
	(0.02)	(0.02)	(0.04)	(0.04)
Interviewer fixed effect	No	Yes	No	Yes
Month effect	No	Yes	No	Yes
Province effect	No	Yes	No	Yes
(Pseduo) *R*^2^	0.051	0.064	0.031	0.041
*N*	6,728	6,728	6,728	6,728

The values in parentheses are robust standard errors of the estimators. ^*^p < 0.10, ^**^p < 0.05, ^***^p < 0.01. Since the data on physical appearance are mainly based on interviewers’ evaluations, and there may be heterogeneity among interviewers, we further control for the interviewer fixed effect.

Among them, Bargain_power is an ordinal variable measuring women’s intra-household bargaining power, Appearance is woman’s physical attractiveness, and X contains control variables. Xnot only includes factors at the individual level, such as household registration, but also factors at the family level, such as spouse’s age. Additionally, it also includes external environmental factors, such as attitudes toward traditional family ethics in community. Monthly and provincial dummies are included in all models to control for seasonal and provincial heterogeneity.

In the third step, to detect a possible causal relationship between physical attractiveness and women’s intra-household bargaining power, we utilize an instrument-variable approach to deal with the endogeneity problem of physical attractiveness (Table 5). There might be endogeneity problem in equation (1), which leads to estimation bias of the coefficient of women’s physical attractiveness. The endogeneity problem mainly comes from the following three aspects. Firstly, there may be a two-way relationship between women’s physical attractiveness and their intra-household bargaining power. That is women’s intra-household bargaining power may adversely affect their physical attractiveness. For example, women with higher intra-household bargaining power may spend more time and money on dressing and grooming, which makes them more physically attractive. Secondly, the interviewers’ subjective evaluations on the respondents’ appearance may be biased. In the process of contacting and communicating, the respondents’ words and behaviors may leave different impressions on the interviewers. This will hinder the interviewers from making objective judgments to some extent. Thirdly, although this paper controls for many important covariates, there may still be problems of omitted variables. Failure to control for unobservable factors that affect both women’s physical attractiveness and their intra-household bargaining power can also lead to bias in coefficient estimates.

**Table 5:** Endogeneity test of physical appearance on women’s intra-household bargaining power.

	(1)	(2)	(3)	(4)
	2SLS		IV-Oprobit	
	First stage	Second stage	First stage	Second stage
*Appearance*		0.28^***^		0.48^***^
		(0.10)		(0.14)
*Appearance_2010*	0.09^***^		0.09^***^	
	(0.01)		(0.01)	
Covariate	Yes	Yes	Yes	Yes
Interviewer fixed effect	Yes	Yes	Yes	Yes
Month effect	Yes	Yes	Yes	Yes
Province effect	Yes	Yes	Yes	Yes
C-D Wald F	87.91		87.91	
AR statistic	7.65^**^		7.65^**^	
Wald $\chi^{2}$		459.03^***^		402.66^***^
N	6,462	6,462	6,462	6,462

C-D Wald F is the Cragg-Donald Wald F value in the first stage; AR statistic is the statistic of weak instrument-variable test; Wald $\chi^{2}$ is the statistical value which judges the overall significance in the second stage model. The values in parentheses are robust standard errors of the estimators. ^*^p < 0.10, ^**^p < 0.05, ^***^p < 0.01. Since the data on physical appearance are mainly based on interviewers’ evaluations, and there may be heterogeneity among interviewers, we further control for the interviewer fixed effect.

In the fourth step, we use the generalized structural equation model (GSEM) to decompose the direct and indirect effects of three mediators between physical attractiveness and women’s intra-household bargaining power (Table 6). Traditional mediating analysis tends to assume a pre- and post-causal relationship^5^ between the three variables, i.e., intervention, mediating, and response variables (Baron and Kenny, 1986). The approach, which sets each variable *a priori* as cause or effect, conflicts with the paradigm that each variable can be either effect or cause in the study of causality. Therefore, the standard regression method is not suitable for directly modeling such a relationship. The structural equation model (SEM), which is popular in psychology and management, provides a more appropriate inferential framework for mediation analysis and other types of causal analysis (Williams et al., 2009; Karimi and Meyer, 2014). Compared with traditional mediation analysis, SEM framework has many advantages in mediation analysis. For example, SEM is easier to estimate and interpret when the model includes latent variables such as happiness, quality of life, and self-esteem. SEM can test many complex mediation models, such as extending the mediation process to multiple interventions, mediation, and response variables. Additionally, Bollen and Pearl (2013) pointed out that standard regression analysis is a statistical relationship based on conditional expected values, while SEM is a functional relationship expressed by conceptual models, path diagrams, and mathematical equations. Hence, the causal relationship in the mediation process, the synchronicity of indirect (or mediation) and direct effects, and the dual role of the mediating variable as the cause of the outcome and the result of the intervention make the use of structural equations more appropriate than the use of regression analysis (Hair et al., 2021).

**Table 6:** Transmission channels.

	(1)	(2)	(3)	(4)	(5)	(6)	(7)
	Income channel		Self-esteem channel		Interpersonal relationship channel		Three channels combined
	*Income*	*Bargaing_power*	*Self_esteem*	*Bargaing_power*	*Relation*	*Bargaing_power*	*Bargaing_power*
*Appearance*	0.06^***^	0.03^**^	0.13^***^	0.03^**^	0.08^***^	0.03^**^	0.03^*^
	(0.02)	(0.02)	(0.02)	(0.02)	(0.01)	(0.02)	(0.02)
*Income*		0.06^***^					0.06^***^
		(0.02)					(0.02)
*Self_esteem*				0.03^***^			0.02^***^
				(0.01)			(0.01)
*Relation*						0.05^**^	0.04^**^
						(0.02)	(0.02)
Covariate	Yes	Yes	Yes	Yes	Yes	Yes	Yes
Interviewer fixed effect	Yes	Yes	Yes	Yes	Yes	Yes	Yes
Month effect	Yes	Yes	Yes	Yes	Yes	Yes	Yes
Province effect	Yes	Yes	Yes	Yes	Yes	Yes	Yes
Indirect effect	0.003^***^		0.003^***^		0.004^**^		0.010^**^
Direct effect	0.030^**^		0.033^**^		0.029^**^		0.030^**^
Mediation effect	10.30%		9.23%		12.02%		33.33%
N	6,728		6,728		6,728		6,728

The values in parentheses are robust standard errors of the estimators. ^*^p < 0.10, ^**^p < 0.05, ^***^p < 0.01. When we put the three mediating variables into a single equation, the respective coefficients of Appearance for Income, Self_esteem and Relation are 0.059(0.015), 0.134(0.022), and 0.083(0.009) with 1% significance level. Since the data on physical appearance are mainly based on interviewers’ evaluations, and there may be heterogeneity among interviewers, we further control for the interviewer fixed effect.

This paper will use the following two structural equation models to estimate the three mediating effects mentioned in the conceptual framework, namely, income, self-esteem, and interpersonal relationship. Where, M is a mediation variable, Appearance is an intervention variable (independent variable in the paper), Bargain_power is a response variable (dependent variable in the paper), and IV is other control variables, including Urban, Education, Age, S_Age, S_Income, S_Relation, and Familylinec.


(2) $$M=\beta_{0}*Appearance+\gamma_{0}*IV+\mu_{m}$$

(3) $$Bargain\_power=\beta_{1}*Appearance+\beta_{2}*M+\gamma_{1}*IV+\mu_{y}$$

As an important assumption for causal inference in mediation analysis, we assume that the error terms $\left(\mu_{m},\mu_{y}\right)$ are uncorrelated. Additionally, we also assume the error terms conform to multivariate normality, a necessary condition for defining direct and indirect (or mediation) effects. The above two structural equations (2) and (3) are related to each other, and their inferences are also carried out simultaneously.^6^ The direct effect is the influencing path of intervention variable Appearance on the response variable Bargain_power when the mediating variable M is controlled, and it is represented by $\beta_{1}$ in equation (3). The indirect (or mediation) effect describes the extent to which the intervention variable Appearance influences the response variable Bargain_power through the mediator M. It is represented by the product of $\beta_{0}$ and $\beta_{2}$.

Considering that the response variable Bargain_power is an ordinal variable, this paper uses generalized SEM (GSEM) to estimate the above equations. The GSEM can estimate the nonlinearly distributed data such as logit, probit, ordinal logit, ordinal probit, multinomial logit, poison, etc., by the generalized linear response model.

In the last step, we replace the dependent variable by using women’s family financial decision-making power to show more robust empirical results in the paper (Table 7).

**Table 7:** The effect of physical attractiveness on women’s financial decision-making power.

	(1)	(2)	(3)	(4)
	2SLS		IV-oprobit	
	First stage	Second stage	First stage	Second stage
*Appearance*		0.26^**^		0.34^**^
		(0.12)		(0.16)
*Appearance_2010*	0.09^***^		0.09^***^	
	(0.01)		(0.01)	
Covariate	Yes	Yes	Yes	Yes
Interviewer fixed effect	Yes	Yes	Yes	Yes
Month effect	Yes	Yes	Yes	Yes
Province effect	Yes	Yes	Yes	Yes
C-D Wald F	88.51		88.51	
AR statistic	4.51^*^		4.51^*^	
Wald $\chi^{2}$		576.17^***^		580.99^***^
N	6,728	6,728	6,728	6,728

The values in parentheses are robust standard errors of the estimators. ^*^p < 0.10, ^**^p < 0.05, and ^***^p < 0.01. Since the data on physical appearance are mainly based on interviewers’ evaluations, and there may be heterogeneity among interviewers, we further control for the interviewer fixed effect.

## Results

### Descriptive analyses

Table 2 lists the descriptive statistics of major variables. According to our calculation, only 15.68% women make major family decisions. In line with the reality of Chinese society, husbands are responsible for major family affairs, while wives play the role of supporting their husbands (Qing, 2020). It is common for husbands to be more educated and earn more than their wives because of son preference and gender discrimination in the workplace (Rozelle et al., 2002; Iwasaki and Ma, 2020). Due to the existence of external traditional family ethics, women in many cases accept the fact that men take the lead in major family affairs (Li, 2021). Additionally, 43.8% of women live in urban areas. The average years of education is 5.91. The average age is 48.51. In terms of gender difference, it is shown that men have more advantages than female counterparts in education and income. Regarding maintaining interpersonal relationships, wives show slightly more advantages than their husbands. In terms of external environment, most of communities show their support for the traditional Chinese family ethics.

Table 3 displays the cross-tabulation of physical attractiveness and women’s intra-household bargaining power. The last row shows that the proportion of wives as decision-makers of major family affairs is much smaller than that of husbands. Additionally, when the score of women’s physical attractiveness increases from 1 to 7, husbands’ dominant position in the family remains unchanged. However, as the score of women’s physical attractiveness increases, so does the proportion of wives who are family decision-makers. For example, when the score of Appearance is 1, the percentage of wives as family decision-makers is 0. While it is increased to 7, the percentage of wives being family decision-makers is 21.27%. Correspondingly, the percentage of husbands as family decision-makers decline from 82.35 to 58.73%, a drop of 23.62%. This suggests a positive correlation between women’s good looks and their intra-household bargaining power.

### Multivariate analyses

#### Regression results

After controlling for relevant covariates, Table 4 displays the direction and extent of the impact of women’s physical attractiveness on their intra-household bargaining power. According to the OLS and ordinal probit regression, Appearance is significantly positive at the 1% level. It indicates that women’s physical attractiveness will enhance their bargaining power in major family affairs. So, Hypothesis 1 is confirmed.

#### Endogeneity problem

To detect any causal relationship, we seek an instrument variable to solve the endogenous problem of physical attractiveness.^7^ Then, we re-estimate the econometric model through two-stage least squares (2SLS) and instrument-variable ordinal probit model (IV-Oprobit). While using physical attractiveness in 2010 as the instrument, the regression results in Table 5 show that the respondents’ physical attractiveness in 2010 is highly correlated with their physical attractiveness in 2012, and the Crag-Donald Wald *F* value in the first stage is greater than 10. So, it excludes the problem of weak instrument variable. In the second stage, physical attractiveness has a positive impact on women’s intra-household bargaining power. The coefficient is greater than the results of OLS and ordinal probit in Table 4. It indicates that the effect of physical attractiveness is likely to be underestimated due to endogeneity problem. In conclusion, the IV-based regression also indicates that women’s physical attractiveness is positively associated with their intra-household bargaining power.

#### Transmission channels

**Figure 2:**
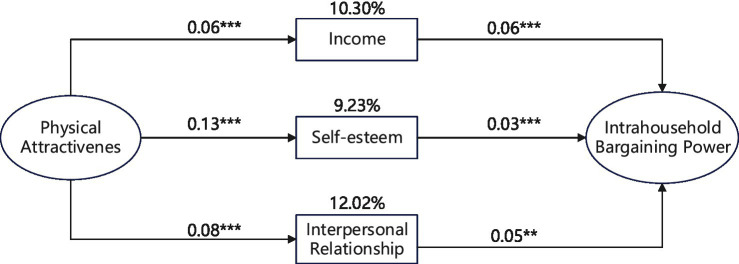
The mediating effects of income, self-esteem, and interpersonal relationship between physical attractiveness and women’s intra-household bargaining power. *p<0.10, **p<0.05, ***p<0.01.

Table 6 and Figure 2 show the GSEM estimation results of intervention, mediation, and response variables. The following conclusions can be drawn. Firstly, the coefficients of Apperancein Columns (1), (3), and (5) are positive, indicating that physical attractiveness can improve women’s income, self-esteem, and interpersonal relationship. Secondly, the coefficients of Income, Self_esteem, and Relation in Columns (2), (4), and (6) are positive, indicating that women’s income, self-esteem, and interpersonal relationship can improve their intra-household bargaining power. Thirdly, when we put the three mediating variables into a single equation, Column (7) shows that income, self-esteem, and interpersonal relationship remain significantly positive.^8^ Fourthly, both direct effects and indirect effects are significant, and the mediation effect for income is 10.30%, self-esteem 9.23%, and interpersonal relationship 12.02%. Therefore, we conclude that physical attractiveness could affect women’s intra-household bargaining power through three channels of income, self-esteem, and interpersonal relationship. Hypothesis 2 is empirically confirmed.

#### Robustness check by replacing the dependent variable

In addition to Bargain_power, we use women’s family financial decision-making power (Finance_power) to measure women’s intra-household bargaining power. Like that in Table 5, we also employ physical attractiveness in 2010 as the instrument variable to address the endogeneity issue. Table 7 displays the regression results. In Columns (1) and (3), F value of Cragg-Donald Wald in the first stage is greater than 10. This excludes the problem of weak instrument variable. In Columns (2) and (4), women’s physical attractiveness can enhance their financial decision-making power in the family. Thus, whether using Bargain_power or Finance_power as the dependent variable, we show physical attractiveness increases women’s bargaining power within the family.

## Discussion and conclusion

Based on the CFPS survey, this paper finds that physical attractiveness significantly enhances women’s intra-household bargaining power. In view of possible endogeneity of physical attractiveness, we employ an instrument-variable-based regression to obtain a similar conclusion. To ensure the robustness of the conclusion, women’s financial decision-making power is constructed as an alternative dependent variable. The regression results corroborate the main findings. Additionally, the influence of covariates on women’s intra-household bargaining power is also consistent with the existing literature (Mabsout and van Staveren, 2010). At the individual level, women living in cities tend to have stronger bargaining power when other things remain equal (Amjad et al., 2021). It may result from more job opportunities and more open and freer social environment (Lu and Song, 2006). Women with more education can compete in the labor market through their acquired knowledge and skills, thus enjoying stronger bargaining power in the family (Moeeni, 2021). Those factors that make husbands more external options when facing the threat of marriage failure can inhibit women’s voices in family affairs. So, husbands with more education and income can indirectly reduce women’s bargaining power in major family affairs (Brown, 2009). The more time and energy a married woman spends on traditional housework (such as child care, laundry, and cooking), the less influence she has on the decision-making process in family affairs (Antman, 2014). Under the influence of traditional sociocultural norms, women are placed in a less important position and subject to the authority of men in making decisions on major family affairs (Agarwal, 1997; Li, 2022). Thus, if women live in a community that emphasizes traditional family ethics, public opinion and moral pressure around can weaken women’s bargaining power within the family.

To examine the influencing channels through which physical attractiveness improves women’s intra-household bargaining power, the generalized structural equation model (GSEM) is used. The results of GSEM show that income, self-esteem, and interpersonal relationship play some important mediating roles between physical attractiveness and women’s intra-household bargaining power. The combined mediating effects of income (10.30%), self-esteem (9.23%), and interpersonal relationship (12.02%) reach up to 33%. The enhancing effect of income on women’s intra-household bargaining power is repeatedly emphasized in the existing literature (Ma and Piao, 2020). Only by raising the income level of women, can they have basic conditions to challenge traditional concept of men being entitled to a place in society that is superior to women. Consequently, it provides the possibility to enhance women’s intra-household bargaining power. Contrastly, personality traits have not been paid enough attention in the literature of women’s development, even though they benefit women greatly in the labor market (Diener and Diener, 2009; Orth and Robins, 2014). Few studies discussed the influence of some factors on women’s intra-household bargaining power from the perspective of women’s personality traits, especially self-esteem (Li and Wang, 2020). Compared with such channels as income and assets, self-esteem traits have the feature of long-term stability (Trzesniewski et al., 2003). Therefore, when the government promotes the idea of gender equality, it also needs to enhance women’s sense of self-esteem through various means, one of which is to cultivate neat appearance.

In the age of valuing appearance, physical attractiveness plays an increasingly important role in individual’s work and life (Hosoda et al., 2006; Ko and Suh, 2019). In addition to labor market, the paper introduces physical attractiveness into the domain of women’s empowerment in the family. The findings imply that as an important way of empowering women, it is encouraged for women to be physically attractive by cultivating own external image and temperament. Of course, here is not to advocate to buy expensive superficial cosmetics, even extreme plastic surgery, but usually pay attention to appearances and behaviors. As the old saying goes in China, “wisdom in hold, elegance in mold,” which emphasizes the comprehensive temperament of speech and manners (Gong et al., 2020). All these can be improved through our day-by-day efforts.

The paper presents some interesting and insightful findings, but there remain some limitations worth noting. Firstly, the time effectiveness of dataset. The data used in the paper were collected in 2012, so, the findings might not reflect the latest situation in China. It is possible that a positive effect of physical attractiveness on women’s intra-household bargaining power is detected in 2012, but such a positive effect would disappear in 2022. Nevertheless, it will be not a big concern, based on recent studies, Chinese families and relation structures have been evolving slowly (Ji and Yeung, 2014; Yu and Xie, 2021). Traditional gender ideologies remain influential in determining both men and women’s roles and behaviors, despite a great socioeconomic transformation (Li, 2021). The esthetic standards have not changed a lot, the pursuit of beauty remains strong (Hua, 2013). Moreover, beauty premium in both social and economic activities does not fall, but it shows an increasing trend (Gu and Ji, 2019; Li et al., 2020). Secondly, generalization of the findings. The paper is focused on the relationship between Chinese women’s physical attractiveness and their bargaining power in the family. So, it is concerning that the findings might not be generalizable to other cultures and countries. Especially, power relations within the family are shaped by cultural norms, conventions, and explicit moral rules (Agarwal, 1997; Madan et al., 2018). According to Global Gender Gap Report,^9^ women enjoy almost equal power with male counterparts in many northern European countries, reflected in household division of labor and expenditure allocation. Regarding this, we should not overgeneralize the findings of the study to other countries. Nevertheless, our findings could provide useful insights to some other countries which share some similar cultural backgrounds, e.g., Eastern Asian countries.

## Data availability statement

Publicly available datasets were analyzed in this study. This data can be found at: https://opendata.pku.edu.cn/dataverse/CFPS?language=en.

## Ethics statement

Informed consent was obtained from all individual participants included in the study.

## Author contributions

The author confirms being the sole contributor of this work and has approved it for publication.

## Funding

This study was funded by the Advance Research Fund for Humanities and Social Sciences of Zhejiang University of Technology, Grant/Award Number: SKY-ZX-20220243; National Natural Science Foundation of China, Grant/Award Number: 42001118; and the Basic Scientific Research Fund of Zhejiang University of Technology, Grant/Award Number: GB202103002.

## Conflict of interest

The author declares that the research was conducted in the absence of any commercial or financial relationships that could be construed as a potential conflict of interest.

## Publisher’s note

All claims expressed in this article are solely those of the authors and do not necessarily represent those of their affiliated organizations, or those of the publisher, the editors and the reviewers. Any product that may be evaluated in this article, or claim that may be made by its manufacturer, is not guaranteed or endorsed by the publisher.

## Footnotes

^1^M￼arket status of China’s cosmetics industry in 2018: The market size for female consumers reaches 2.9 trillion yuan, and beauty and skin care consumption dominates, 05, Sep, 2018, Retrieved from https://www.askci.com/news/chanye/20180905/1748071131196.shtml

^2^That is, physically attractive persons earn 10–15% more income than less physically attractive counterparts.

^3^In the CFPS, every respondent is assigned with a unique PID, with which we can continuously track the respondent in next several rounds of surveys.

^4^The method of instrumental variables (IV) is used to estimate causal relationships when controlled experiments are not feasible or when a treatment is not successfully delivered to every unit in a randomized experiment.

^5^It sets each variable *a priori* as cause or effect.

^6^It means we make inferences on parameters of interests in both equations (2) and (3) together.

^7^In econometrics, endogeneity broadly refers to situations in which an independent variable is correlated with the error term. The problem of endogeneity will make biased estimations of coefficient of interests in non-experimental research. The Instrumental variable techniques are commonly used to address this problem.

^8^When we put the three mediating variables into a single equation, the respective coefficients of Appearance ￼for Income￼, Self_esteem ￼and Relation ￼are 0.059(with standard error of 0.015), 0.134(with standard error of 0.022), 0.083(with standard error of 0.009) with 1% significance level. We do not display the results in a separate Column for sake of saving space.

^9^2022 Global Gender Gap Report, Available at: https://www.weforum.org/reports/global-gender-gap-report-2022/

## References

[ref1] Agarwal B. (1997). “Bargaining” and gender relations: within and beyond the household. Fem. Econ. 3, 1–51. doi: 10.1080/135457097338799

[ref2] Amjad M. Nawaz A. Anwar M. M. Farooq A. (2021). The role of socio-economic factors in determining the women bargaining power in Pakistan. J. Bus. Soc. Rev. Emerg. Econ. 7, 467–480. doi: 10.26710/jbsee.v7i2.1823

[ref3] Andreoni J. Petrie R. (2008). Beauty, gender and stereotypes: evidence from laboratory experiments. J. Econ. Psychol. 29, 73–93. doi: 10.1016/j.joep.2007.07.008

[ref4] Antman (2014). Spousal employment and intra-household bargaining power. Appl. Econ. Lett. 21, 560–563. doi: 10.1080/13504851.2013.875101, PMID: 25342928 PMC4203440

[ref5] Anýžová P. Matějů P. (2018). Beauty still matters: the role of attractiveness in labour market outcomes. Int. Sociol. 33, 269–291. doi: 10.1177/0268580918760431

[ref6] Baland J.-M. Ziparo R. (2018). “Intra-household bargaining in poor countries,” in Towards Gender Equity in Development. eds. Anderson S. Beaman L. Platteau J.-P. (Oxford, England: Oxford University Press).

[ref7] Baron R. A. (2000). Psychological perspectives on entrepreneurship: cognitive and social factors in Entrepreneurs' success. Curr. Dir. Psychol. Sci. 9, 15–18. doi: 10.1111/1467-8721.00050

[ref8] Baron R. M. Kenny D. A. (1986). The moderator–mediator variable distinction in social psychological research: conceptual, strategic, and statistical considerations. J. Pers. Soc. Psychol. 51, 1173–1182. doi: 10.1037/0022-3514.51.6.1173, PMID: 3806354

[ref9] Bénabou R. Tirole J. (2002). Self-confidence and personal motivation. Q. J. Econ. 117, 871–915. doi: 10.1162/003355302760193913

[ref10] Bollen K. A. Pearl J. (2013). “Eight myths about causality and structural equation models,” in Handbook of Causal Analysis for Social Research. ed. Morgan S. L. (New York: Springer), 301–328.

[ref11] Bowles S. Gintis H. Osborne M. (2001). Incentive-enhancing preferences: personality, behavior, and earnings. Am. Econ. Rev. 91, 155–158. doi: 10.1257/aer.91.2.155

[ref12] Brand R. J. Bonatsos A. D’Orazio R. DeShong H. (2012). What is beautiful is good, even online: correlations between photo attractiveness and text attractiveness in men’s online dating profiles. Comput. Hum. Behav. 28, 166–170. doi: 10.1016/j.chb.2011.08.023

[ref13] Brown P. (2009). Dowry and Intrahousehold bargaining: evidence from China. J. Hum. Resour. 44, 25–46. doi: 10.2139/ssrn.444820

[ref14] Datta Gupta N. Etcoff N. L. Jaeger M. M. (2016). Beauty in mind: the effects of physical attractiveness on psychological well-being and distress. J. Happiness Stud. 17, 1313–1325. doi: 10.1007/s10902-015-9644-6

[ref15] Deng W. Li D. Zhou D. (2020). Beauty and job accessibility: new evidence from a field experiment. J. Popul. Econ. 33, 1303–1341. doi: 10.1007/s00148-019-00744-7

[ref16] Diener E. Diener M. (2009). “Cross-cultural correlates of life satisfaction and self-esteem” in Culture and Well-Being: The Collected Works of Ed Diener. ed. Diener E. (Netherlands: Springer), 71–91.

[ref17] Dilmaghani M. (2021). Sitting pretty: satisfaction with physical appearance, division of household chores, and satisfaction with housework. Soc. Sci. J. 1-24, 1–24. doi: 10.1080/03623319.2021.1922978

[ref18] Dion K. Berscheid E. Walster E. (1972). What is beautiful is good. J. Pers. Soc. Psychol. 24, 285–290. doi: 10.1037/h0033731 4655540

[ref19] Eckel C. Wilson R. (2004). Detecting trustworthiness: does beauty confound intuition? (Discussion paper, Issue).

[ref20] Eklund P. Imai K. Felloni F. (2007). Women's organisations, maternal knowledge, and social capital to reduce prevalence of stunted children: evidence from rural Nepal. J. Dev. Stud. 43, 456–489. doi: 10.1080/00220380701204406

[ref21] Esping-Andersen G. Schmitt C. (2019). Multi-dimensional couple bargaining and housework allocation. Acta Sociol. 63, 3–22. doi: 10.1177/0001699319859418

[ref22] Frieze I. H. Olson J. E. Good D. C. (1990). Perceived and actual discrimination in the salaries of male and female managers. J. Appl. Soc. Psychol. 20, 46–67. doi: 10.1111/j.1559-1816.1990.tb00377.x

[ref23] Gong P. Tao Y. Wang H. Cao K. Peng Y. (2020). Research on psychological management problems of military cadets under the angle of group psychology. Man-Machine-Environment System Engineering, Singapore.

[ref24] Gordon R. A. Crosnoe R. Wang X. (2013). Physical attractiveness and the accumulation of social and human capital in adolescence and young adulthood: assets and distractions. Monogr. Soc. Res. Child Dev. 78, 1–137. doi: 10.1002/mono.12060, PMID: 24329915 PMC5558203

[ref25] Grammer K. Fink B. Møller A. P. Thornhill R. (2003). Darwinian aesthetics: sexual selection and the biology of beauty. Biol. Rev. Camb. Philos. Soc. 78, 385–407. doi: 10.1017/s1464793102006085, PMID: 14558590

[ref26] Gray-Little B. Williams V. S. L. Hancock T. D. (1997). An item response theory analysis of the Rosenberg self-esteem scale. Personal. Soc. Psychol. Bull. 23, 443–451. doi: 10.1177/0146167297235001

[ref27] Gu T. Ji Y. (2019). Beauty premium in China's labor market: is discrimination the main reason? China Econ. Rev. 57:101335. doi: 10.1016/j.chieco.2019.101335

[ref28] Haas A. Gregory S. W. (2005). The impact of physical attractiveness on Women's social status and interactional power. Sociol. Forum 20, 449–471. doi: 10.1007/s11206-005-6597-2

[ref29] Hair J. F. Hult G. T. M. Ringle C. M. Sarstedt M. Danks N. P. Ray S. (2021). “An introduction to structural equation modeling” in Partial Least Squares Structural Equation Modeling (PLS-SEM) Using R: A Workbook. eds. Hair J. F. Jr. Hult G. T. M. Ringle C. M. Sarstedt M. Danks N. P. Ray S. (Cham, Switzerland: Springer International Publishing), 1–29.

[ref30] Hamermesh D. S. Abrevaya J. (2013). Beauty is the promise of happiness? Eur. Econ. Rev. 64, 351–368. doi: 10.1016/j.euroecorev.2013.09.005

[ref31] Hamermesh D. S. Biddle J. E. (1994). Beauty and the labor market. Am. Econ. Rev. 84, 1174–1194.

[ref32] Hatfield E. Sprecher S. (1986). The Importance of Looks in Everyday Life. Albany, New York: State University of New York Press.

[ref33] Heckman J. Stixrud J. Urzua S. (2006). The effects of cognitive and noncognitive abilities on labor market outcomes and social behavior. J. Labor Econ. 24, 411–482. doi: 10.1086/504455

[ref34] Hosoda M. Stone-Romero E. F. Coats G. (2003). The effects of physical attractiveness on job-related outcomes: a meta-analysis of experimental studies. Pers. Psychol. 56, 431–462. doi: 10.1111/j.1744-6570.2003.tb00157.x

[ref35] Hosoda M. Stone-Romero E. Coats G. (2006). The effects of physical attractiveness on job-related outcomes: a meta-analysis of experimental studies. Pers. Psychol. 56, 431–462. doi: 10.1111/j.1744-6570.2003.tb00157.x

[ref36] Hua W. E. N. (2009). “Being Good-looking is capital”: cosmetic surgery in China today. Asian Anthropol. 8, 89–107. doi: 10.1080/1683478X.2009.10552588

[ref37] Hua W. (2013). Buying Beauty: Cosmetic Surgery in China. Hongkong: Hong Kong University Press.

[ref38] Ivtzan I. Moon H. S. (2008). The beauty of self-actualisation: linking physical attractiveness and self-fulfilment. Europe’s. Aust. J. Psychol. 4, 34–46. doi: 10.5964/ejop.v4i4.439

[ref39] Iwasaki I. Ma X. (2020). Gender wage gap in China: a large meta-analysis. J. Lab. Market Res. 54:17. doi: 10.1186/s12651-020-00279-5

[ref40] Jackson L. A. Hunter J. E. Hodge C. N. (1995). Physical attractiveness and intellectual competence: a meta-analytic review. Soc. Psychol. Q. 58, 108–122. doi: 10.2307/2787149

[ref41] Ji Y. Yeung W.-J. J. (2014). Heterogeneity in contemporary Chinese marriage. J. Fam. Issues 35, 1662–1682. doi: 10.1177/0192513X14538030

[ref42] Jin J. Fan B. Dai S. Ma Q. (2017). Beauty premium: event-related potentials evidence of how physical attractiveness matters in online peer-to-peer lending. Neurosci. Lett. 640, 130–135. doi: 10.1016/j.neulet.2017.01.037, PMID: 28111351

[ref43] Judge T. A. Hurst C. Simon L. S. (2009). Does it pay to be smart, attractive, or confident (or all three)? Relationships among general mental ability, physical attractiveness, Core self-evaluations, and income. J. Appl. Psychol. 94, 742–755. doi: 10.1037/a0015497, PMID: 19450010

[ref44] Kabeer N. (1999). Resources, agency, achievements: reflections on the measurement of Women's empowerment. Dev. Chang. 30, 435–464. doi: 10.1111/1467-7660.00125

[ref45] Kanazawa S. (2011). Intelligence and physical attractiveness. Intelligence 39, 7–14. doi: 10.1016/j.intell.2010.11.003

[ref46] Karimi L. Meyer D. (2014). Structural equation modeling in psychology: the history, development and current challenges. Int. J. Psychol. Stud. 6, 123–133. doi: 10.5539/ijps.v6n4p123

[ref47] Ko A. Suh E. M. (2019). Does physical attractiveness buy happiness? Women’s mating motivation and happiness. Motiv. Emot. 43, 1–11. doi: 10.1007/s11031-018-9717-z

[ref48] Lai E. Wolfe E. Vickers D. (2013). Revisiting the halo effect within a multitrait. Multimethod Framework Annual meeting of the American Educational Research Association, San Francisco, CA.

[ref49] Langlois J. H. Kalakanis L. Rubenstein A. J. Larson A. Hallam M. Smoot M. (2000). Maxims or myths of beauty? A meta-analytic and theoretical review. Psychol. Bull. 126, 390–423. doi: 10.1037//0033-2909.126.3.390 10825783

[ref50] Lee L. Loewenstein G. Ariely D. Hong J. Young J. (2008). If I'm not hot, are you hot or not?: physical-attractiveness evaluations and dating preferences as a function of One's own attractiveness. Psychol. Sci. 19, 669–677. doi: 10.1111/j.1467-9280.2008.02141.x 18727782

[ref51] Li Z. (2021). Does family decision-making power improve Women’s happiness? J. Fam. Issues 43, 2016–2039. doi: 10.1177/0192513X211030025

[ref52] Li Z. (2022). Does intrahousehold bargaining power enhance women's marital satisfaction? A perspective from two competing forces in China. Rev. Dev. Econ. doi: 10.1111/rode.12947 [Epub ahead of print].

[ref53] Li C. Lin A.-P. Lu H. Veenstra K. (2020). Gender and beauty in the financial analyst profession: evidence from the United States and China. Rev. Acc. Stud. 25, 1230–1262. doi: 10.1007/s11142-020-09542-z

[ref54] Li Z. Wang Q. (2020). Ti Gao nv xing jia ting di wei de xin li tu jing: Zi wo ren tong de li zi a [Psychological Channel to improve Women’S family Status: A Case of Self-esteem]. Statis. Res. 37, 44–56. doi: 10.19343/j.cnki.11-1302/c.2020.11.004

[ref55] Lu Z. Song S. (2006). Rural–urban migration and wage determination: the case of Tianjin, China. China Econ. Rev. 17, 337–345. doi: 10.1016/j.chieco.2006.04.007

[ref56] Lynn M. J. A. O. S. B. (2009). Determinants and consequences of female attractiveness and sexiness. Real. Tests Restaur. Waitress. 38, 737–745. doi: 10.1007/s10508-008-9379-0, PMID: 18543091

[ref57] Ma X. Piao X. (2020). “Income, intra-household bargaining power and the happiness of Japanese married women” in Quality of Life in Japan: Contemporary Perspectives on Happiness. eds. Tsai M.-C. Iwai N. (Singapore: Springer Singapore), 77–106.

[ref58] Mabsout R. van Staveren I. (2010). Disentangling bargaining power from individual and household level to institutions: evidence on Women’s position in Ethiopia. World Dev. 38, 783–796. doi: 10.1016/j.worlddev.2009.11.011

[ref59] Madan S. Basu S. Ng S. Lim E. (2018). Impact of culture on the pursuit of beauty: evidence from five countries. J. Int. Mark. 26, 54–68. doi: 10.1509/jim.17.0064

[ref60] Major B. Carrington P. I. Carnevale P. J. D. (1984). Physical attractiveness and self-esteem: attributions for praise from an other-sex evaluator. Personal. Soc. Psychol. Bull. 10, 43–50. doi: 10.1177/0146167284101004

[ref61] Martínez C. (2013). Intrahousehold allocation and bargaining power: evidence from Chile. Econ. Dev. Cult. Chang. 61, 577–605. doi: 10.1086/669260

[ref62] Mayoux L. (2001). Tackling the down side: social capital. Women’s Empower. Micro-Fin. Cameroon. 32, 435–464. doi: 10.1111/1467-7660.00212

[ref63] Mobius M. M. Rosenblat T. S. (2006). Why beauty matters. Am. Econ. Rev. 96, 222–235. doi: 10.1257/000282806776157515

[ref64] Moeeni S. (2021). Married women’s labor force participation and intra-household bargaining power. Empir. Econ. 60, 1411–1448. doi: 10.1007/s00181-019-01800-7

[ref65] O’Connor K. M. Gladstone E. (2018). Beauty and social capital: being attractive shapes social networks. Soc. Networks 52, 42–47. doi: 10.1016/j.socnet.2017.05.003

[ref66] Oreffice S. Quintana-Domeque C. (2012). Fat spouses and hours of work: are body and Pareto weights correlated? IZA J. Labor Econ. 1:6. doi: 10.1186/2193-8997-1-6

[ref67] Orth U. Robins R. W. (2014). The development of self-esteem. Curr. Dir. Psychol. Sci. 23, 381–387. doi: 10.1177/0963721414547414

[ref68] Orth U. Robins R. W. Roberts B. W. (2008). Low self-esteem prospectively predicts depression in adolescence and young adulthood. J. Pers. Soc. Psychol. 95, 695–708. doi: 10.1037/0022-3514.95.3.695, PMID: 18729703

[ref69] Patzer G. L. (2006). The Power and Paradox of Physical Attractiveness. Boca Raton, Florida: Universal Publishers.

[ref70] Peng L. Wang X. Ying S. (2020). The heterogeneity of beauty premium in China: evidence from CFPS. Econ. Model. 90, 386–396. doi: 10.1016/j.econmod.2019.12.014

[ref71] Póvoa A. C. S. Pech W. Viacava J. J. C. Schwartz M. T. (2020). Is the beauty premium accessible to all? An experimental analysis. J. Econ. Psychol. 78:102252. doi: 10.1016/j.joep.2020.102252

[ref72] Qing S. (2020). Gender role attitudes and male-female income differences in China. J. Chin. Soc. 7:12. doi: 10.1186/s40711-020-00123-w

[ref73] Rozelle S. Dong X.-Y. Zhang L. Mason A. (2002). Gender wage gaps in post-reform rural China. Pac. Econ. Rev. 7, 157–179. doi: 10.1111/1468-0106.00009

[ref74] Sainani K. L. (2015). Q & amp; A: Karl Grammer. Nature 526:S11. doi: 10.1038/526S11a 26444367

[ref75] Scholz J. Sicinski K. (2015). Facial attractiveness and lifetime earnings: evidence from a cohort study. Rev. Econ. Stat. 97, 14–28. doi: 10.1162/REST_a_00435, PMID: 30505018 PMC6261420

[ref76] Tafarodi R. W. Swann W. B. Jr. (1995). Self-liking and self-competence as dimensions of global self-esteem: initial validation of a measure. J. Pers. Assess. 65, 322–342. doi: 10.1207/s15327752jpa6502_8, PMID: 8656329

[ref77] Tartaglia S. Rollero C. (2015). The effects of attractiveness and status on personality evaluation. Eur. J. Psychol. 11, 677–690. doi: 10.5964/ejop.v11i4.896, PMID: 27247685 PMC4873083

[ref78] Thornton B. Ryckman R. M. (1991). Relationship between physical attractiveness, physical effectiveness, and self-esteem: a cross-sectional analysis among adolescents. J. Adolesc. 14, 85–98. doi: 10.1016/0140-1971(91)90047-U, PMID: 2050868

[ref79] Tran A. Rosales R. Copes L. (2020). Paint a better mood? Effects of makeup use on you tube beauty influencers’ self-esteem. SAGE Open 10:215824402093359. doi: 10.1177/2158244020933591

[ref80] Trzesniewski K. H. Donnellan M. B. Robins R. W. (2003). Stability of self-esteem across the life span. J. Pers. Soc. Psychol. 84, 205–220. doi: 10.1037/0022-3514.84.1.205, PMID: 12518980

[ref81] Tsiga E. Panagopoulou E. Benos A. (2016). Patient attractiveness reduces the likelihood of a missed diagnosis: implications for person-centered healthcare. Eur. J. Pers. Cent. Healthc. 4:439. doi: 10.5750/ejpch.v4i3.1098

[ref82] Waiyee Y. (2021). Plastic surgery booming in China despite the dangers. BBC News. Available at: https://www.bbc.com/news/world-asia-china-57691525 (Accessed October 20, 2021).

[ref83] Wang S.-Y. (2014). Property rights and intra-household bargaining. J. Dev. Econ. 107, 192–201. doi: 10.1016/j.jdeveco.2013.12.003

[ref84] Wang Z. Lou Y. Zhou Y. (2020). Bargaining power or specialization? Determinants of household decision making in Chinese rural migrant families. SAGE Open 10:215824402098044. doi: 10.1177/2158244020980446

[ref85] Williams L. J. Vandenberg R. J. Edwards J. R. (2009). Structural equation modeling in management research: a guide for improved analysis. Acad. Manag. Ann. 3, 543–604. doi: 10.1080/19416520903065683

[ref86] Wong J. S. Penner A. M. (2016). Gender and the returns to attractiveness. Res. Soc. Stratific. Mob. 44, 113–123. doi: 10.1016/j.rssm.2016.04.002

[ref87] Xie Y. Hu J. (2014). An introduction to the China family panel studies (CFPS). Chin. Sociol. Rev. 47, 3–29. doi: 10.2753/CSA2162-0555470101.2014.11082908

[ref88] Xie Y. Lu P. (2015). The sampling design of the China family panel studies (CFPS). Chin. J. Sociol. 1, 471–484. doi: 10.1177/2057150X15614535, PMID: 29854418 PMC5973535

[ref89] Yu J. Xie Y. (2021). Recent trends in the Chinese family: national estimates from 1990 to 2010. Demogr. Res. 44, 595–608. doi: 10.4054/DemRes.2021.44.25

